# Does Relative Age Affect Career Length in North American Professional Sports?

**DOI:** 10.1186/s40798-016-0042-3

**Published:** 2016-01-15

**Authors:** C. Steingröver, N. Wattie, J. Baker, J. Schorer

**Affiliations:** 1 Department of Sport and Motion, Institute of Sport Science, University of Oldenburg, Uhlhornsweg 49-55, 26129 Oldenburg, Germany; 2 Faculty of Health Sciences, University of Ontario Institute of Technology, Oshawa, Canada; 3 Department of Kinesiology and Health Science, York University, Toronto, Canada

## Abstract

**Background:**

Relative age effects (RAEs) typically favour older members within a cohort; however, research suggests that younger players may experience some long-term advantages, such as longer career length. The purposes of this study were to replicate previous findings on RAEs among National Hockey League (NHL) ice hockey players, National Basketball Association (NBA) basketball players and National Football League (NFL) football players and to investigate the influence of relative age on career length in all three sports.

**Methods:**

Using official archives, birthdates and number of games played were collected for players drafted into the NBA (*N* = 407), NFL (*N* = 2380) and NHL (*N* = 1028) from 1980 to 1989. We investigated the possibility that younger players might be able to maximize their career length by operationalizing career length as players’ number of games played throughout their careers.

**Results:**

There was a clear RAE for the NHL, but effects were not significant for the NBA or NFL. Moreover, there was a significant difference in matches played between birth quartiles in the NHL favouring relatively younger players. There were no significant quartiles by career length effects in the NBA or NFL.

**Conclusions:**

The significant relationship between relative age and career length provides further support for relative age as an important constraint on expertise development in ice hockey but not basketball or football. Currently, the reason why relatively younger players have longer careers is not known. However, it may be worth exploring the influence of injury risk or the development of better playing skills.

## Key Points

Results support the notion that relative age is an important constraint on the development of expertise in ice hockey.

A significant relationship between relative age and career length was found in ice hockey.

Relative age did not influence career length of NBA or NFL players in the current study, providing further support that relative age is not relevant to the development of expertise in these sports.

## Background

In many sports, a competition is organized using cut-off dates which results in specific selection, participation and attainment disadvantages due, hypothetically, to the range of physical and cognitive variability within the age cohort (cf. [1–4]). As a result, relative age effects (RAEs) are a widespread phenomenon (for reviews, see [1, 2, 4, 5]). During the last three decades, researchers have identified overrepresentations of athletes born in the first quartile of the selection year (i.e. January to March if the cut-off date is 1 January) across cultural contexts in sports such as football [6–8], ice hockey [9–11], handball [12–14], baseball [15], basketball [16, 17], rugby [18, 19], volleyball [20], tennis [21], ski sports [22] and swimming [23]. Till et al. [19] demonstrated the possible extent of such overrepresentations of relatively older players in rugby: 47.0 % of the regional and 55.7 % of the national junior representative players were born in the first 3 months of the selection year. Overall, uneven birthdate distributions were found continuously from the earliest stages of game involvement up to senior professional levels, with the chance of a RAE increasing with each competition level [19].

Researchers have proposed two main hypotheses to explain the prevalence of RAEs in sports. The *maturation hypothesis* suggests that, especially in younger levels of competition, relative age differences lead to significant differences in height and weight between individuals of the same age cohort. Relatively older players benefit from their greater maturational status because physical advantages may result in superior performance and better opportunities for competition compared with their younger less physically developed teammates [5]. Consequently, a youth athlete’s likelihood of being identified as ‘talented’ [24] and of being selected for higher levels of competition [25] is higher if they are the relatively oldest in their cohort. The *selection hypothesis* considers the very first selection processes as crucial events in an athlete’s development. Once selected, relatively older athletes may profit from better coaches and improved training conditions, greater practice time, higher and more intense competition and more positive and constructive feedback [5, 26].

As noted before, in early levels of selection, relatively older athletes may be favoured because of their advantages in height and strength. These advantages result in significant overrepresentations of players born shortly after the cut-off date. However, Schorer et al. [14] suggested that this trend changes over time; in male German national team handball players, the relatively youngest players made up a larger percentage of the players. There is some evidence to suggest that across development, relatively younger athletes may benefit from their ‘disadvantages’ compared to older peers. Evidence in Canadian ice hockey [27] suggests it might be advantageous to be relatively younger in the adult period of one’s sporting career. The authors examined whether relative age influenced when players were drafted to play in the National Hockey League (NHL), an important step towards a professional ice hockey career and an indicator of their perceived athletic potential. Surprisingly, their data showed that relatively younger athletes were more likely to be chosen in earlier rounds of the draft suggesting they are more sought-after than relatively older players.

Ashworth and Heyndels [28] also demonstrated long-term positive outcomes for relatively younger players. In German soccer, late-born (i.e. in the second half of the selection year) professional players were shown to have higher wages compared to relatively older players. More recently, Hancock et al.’s [29] study of Canadian regional junior female gymnasts also suggests that being relatively younger does not necessarily have a detrimental effect on an athlete’s development. The authors found out that after the age of 14, a reversed RAE occurred so that relatively younger gymnasts were more likely to compete in higher levels of competition and to participate in representative teams [29]. Lastly, Deaner, Lowen and Cobley [30] analysed NHL drafts from 1980 to 2006. They suggested that the players born in the second half of the selection year were drafted later than their older counterparts born in quartile 1 and that the relatively younger players were significantly more likely to reach notable career milestones (e.g. 400 games played or 200 points scored). Ashworth and Heyndels [28] hypothesized that players born closer to the end of the selection year may represent a psychologically resilient minority of their age group that have been able to cope with the disadvantageous selection processes in the scouting system. It is also possible that relatively younger athletes may have benefited from superior practice and development systems that enabled them to benefit from training and competing with better or more mature teammates [25]. Although the precise reason why relatively younger players have more positive outcomes later in their athlete career is not known (e.g. [12]), this emerging evidence suggests that relative age-determined advantages and disadvantages likely change over the course of an athlete’s development.

The current study considered whether relative age influenced career length. Given the variability in athletes’ career length across different sports and different cultures, we sought an appropriate context for comparison where basic organizational characteristics of the different sports and the environmental context would be comparable. Moreover, we looked for a sporting nation where some variability in RAEs have been found across various sports. In the end, we focused on the rich team sport landscape of North America, which has shown team sports with strong and medium RAEs (various levels of ice hockey; [10, 27]) as well as team sports without RAEs (e.g. American football; [31]). RAEs have often been related to sports in which height and strength are said to play an important role in performance; however, Côté, MacDonald, Baker and Abernethy’s [32] examination of birthdates among American players in the National Basketball Association (NBA) found no evidence of a RAE, which is contrary to studies of basketball players in France and Australia [16, 17, 33]. Although RAEs have not been observed in overall samples of American football and basketball players, research has yet to consider whether or not athletes of different relative age, despite their equal representation, experience varying levels of success which is eventually reflected in higher numbers of games played (i.e. career lengths).

With these diverse results in mind, the aims of our study were to replicate previous findings on RAEs among NHL ice hockey players, NBA basketball players and NFL football players and in a second step to investigate the influence of relative age on career length in all three sports. These sports were chosen because the North American leagues are arguably the most elite leagues internationally. Based on the studies presented above [9, 14], we hypothesized that relatively younger athletes would have longer careers than relatively older ones and that these results would be most pronounced in ice hockey since the strongest RAEs have been found in this sport compared to American football (now referred to simply as football) and basketball. Furthermore, we considered whether relative age was related to a higher number of matches played at the highest levels of sport achievement. We hypothesized that in sports with RAEs, those born in quartile 4 will have more matches than their relatively older teammates.

## Methods

### Participants

The sample included players drafted into the NBA (*N* = 407), NFL (*N* = 2380) and NHL (*N* = 1028) from 1980 to 1989 who participated in one or more matches. These cohorts were chosen because most players (>99.9 %) drafted during this decade had finished their active career by the time of our data collection. This ensured that players’ data were comprised of complete career statistics and the total number of games played within their career. Given that the number of players who had not ceased their active career was less than 0.03 % of the sample (i.e. one player), we did not consider them likely to distort our analysis.

### Data Collection

The career statistics were collected through the official websites of the professional associations (www.nhl.com, www.nfl.com and www.nba.com). These online resources contain links to players’ individual statistics and information regarding every year’s draft for each respective sport. Although regarded as reliable, the data collected from the websites were tested for reliability by comparing a random selection of 10 % of the players with official encyclopaedias of North American professional sports (e.g. [34, 35]). There was 100 % consistency between these sources.

### Statistical Analyses

The structure of our statistical analyses was consistent with our three hypotheses. First, we investigated whether RAEs existed across the three sports. To test for RAEs, each player’s birth month was recoded to reflect his birth quartile. The calendar year for those North American sports is from 1 January to 31 December, and accordingly quartile 1 = January, February and March; quartile 2 = April, May and June; quartile 3 = July, August and September; and quartile 4 = October, November and December. As with much of previous research in this area, comparisons were based on the assumption of equal distributions across the quartiles (cf. [12]). Cobley et al.’s study [1] suggested this approach, while not absolutely accurate was in fact a more conservative approach, potentially decreasing the risk of type I error ([1], p. 239). Chi-square tests were administered, and *χ*^2^ values, probabilities (*p*) and effect sizes (*w*) reported. Where chi-square tests were statistically significant, post hoc odds ratio (OR) and 95 % confidence intervals (95 % CI) were calculated to provide additional information about the size and direction of the observed RAE (by comparing the odds of quartile 1 (Q1) to the odds of quartile 4 (Q4)).

Concerning our second hypothesis regarding differences between birthdate quartiles for total matches played, we conducted a unidirectional one factorial analysis of variance with birth quartile as the independent variable and total matches played as the dependent variable. Here, we report *F* values, probabilities (*p*) and effect sizes (*f*).

Our third hypothesis was more exploratory. We wanted to investigate whether any of the outer quartiles (Q1 or Q4) would show a higher number of matches played. We refrained from conducting inferential analyses and instead present a series of graphical evaluations that show the four separate plots for the quartiles with number of games played (*y*-axis) relative to the ranking of an athlete in his birth quartile in our sample. The players were ranked by number of games played so that the players with the most games in his quartile would be on position 1 (*x*-axis), the player with the second highest on position 2 and so on [36]. All analyses were conducted with SPSS 21.0 and MS Excel 2010. Effect sizes were calculated using G*power 3.1.10 [37].

## Results

**Fig. 1:**
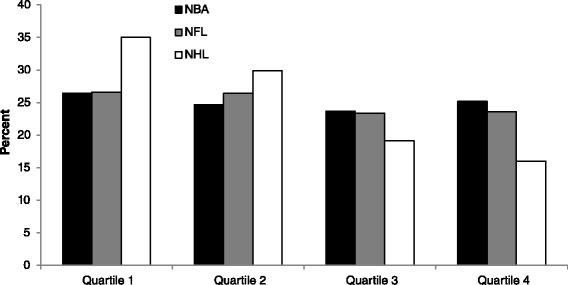
Birthdate distribution within the North American sports in percent (*NBA* National Basketball Association, *NFL* National Football League, *NHL* National Hockey League)

In a first step, we wanted to replicate previous findings on RAEs in all three sports. As can be seen in Fig. 1, in all sports, quartile 1 was overrepresented in comparison to the other quartiles. There was a clear RAE for the NHL sample, *χ*^2^(3, *n* = 700) = 66.89, *p* < .01, *w* = .31. Post hoc analysis suggested that the ratio of the odds of NHL athletes being in Q1 vs. Q4 was approximately two times greater (OR 2.19, 95 % CI 1.61–2.97) compared to the odds of being born in Q1 vs. Q4 in an equally distributed sample. Although there was a trend for the effect, it was not statistically significant for the NBA data, *χ*^2^(3, *n* = 535) = 0.77, *p* = .86, *w* = .04, and only approached significance for the NFL, *χ*^2^(3, *n* = 1924) = 7.03, *p* = .07, *w* = .06.

For our second hypothesis, a one-tailed ANOVA with number of games as dependent variable and quartile as between subject factor was calculated to test for differences in career length in all three sports. Again, a significant difference in matches played between birth quartiles was only found in the NHL, *F*(3, 696) = 2.07, *p* = .05, *f* = .10. As can be seen in Table 1, the players from quartile 4 had the most games followed by quartile 3, quartile 2 and quartile 1. For the NBA, no significant differences was found between the quartiles, *F*(3, 531) = 0.78, *p* = .51, *f* < .01, although quartile 4 had the highest mean of games played. The same pattern of results was found for the NFL sample, *F*(3, 1920) = 0.28, *p* = .84, *f* < .01.

**Table 1:** Mean numbers of games played per quartile and sport (=standard deviations)

	Quartile 1	Quartile 2	Quartile 3	Quartile 4
NBA	340.6 (357.7)	371.5 (374.9)	364.6 (369.7)	408.9 (398.6)
NFL	65.0 (60.2)	64.4 (59.7)	65.6 (57.7)	67.7 (63.4)
NHL	313.7 (359.3)	366.6 (424.8)	394.9 (424.6)	410.9 (402.6)

*NBA* National Basketball Association, *NFL* National Football League, *NHL* National Hockey League

**Fig. 2:**
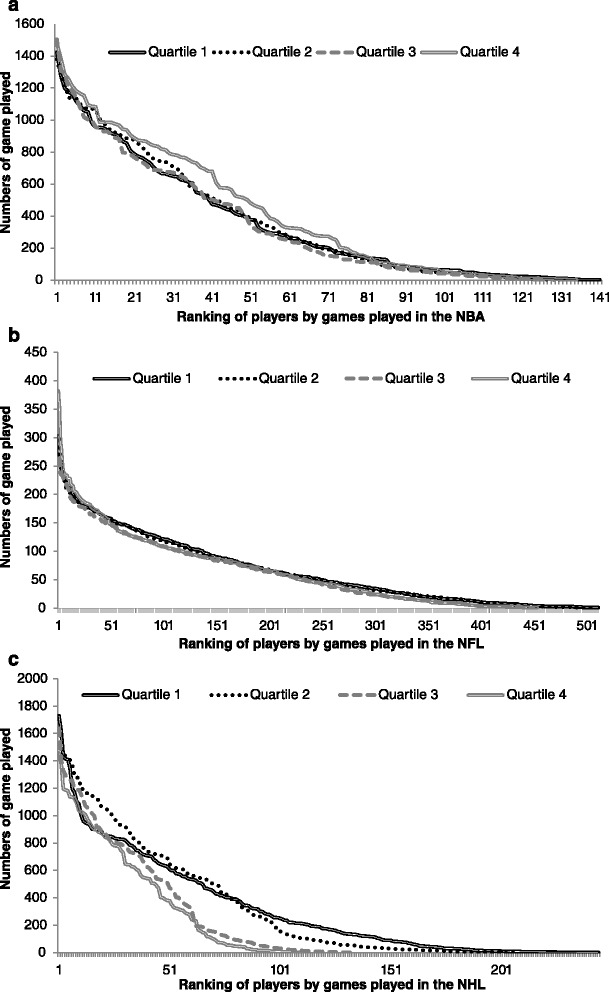
**a**–**c** NBA, NFL and NHL, respectively (*NBA* National Basketball Association, *NFL* National Football League, *NHL* National Hockey League). **a** Ranking of players by games played in the NBA **b** Ranking of players by games played in the NFL. **c** Ranking of players by games played in the NHL

To explore the differences between quartiles in relation to numbers of games played (*y*-axis) and ranking of players by games played (*x*-axis), we used a graphical approach [36, 38, 39]. In the NBA sample (Fig. 2a), quartile 4 outperformed the others in number of games played approximately for the ranks 25 to 75. Above and below those ranks, performance seems to be almost equal. For the NFL, no clear differences were revealed (cf. Fig. 2b). For the NHL, an opposite trend to the NBA occurred. Here, players born in quartiles 1 and 2 seemed to outperform the others approximately in the ranks 25 to 125 (Fig. 2c).

## Discussion

The current study explored the relationship between relative age and athletes’ career length in three prominent North American sports, namely ice hockey, basketball and football. Importantly, we chose a sample that almost eliminated the possibility of including athletes whose careers were still ongoing and therefore were still contributing to career-playing statistics. We hypothesized that in sports where RAEs are found, relatively younger players would have longer careers and that these effects would be most pronounced in ice hockey. Findings suggested that there was no tangible relationship between relative age and career length among the samples of professional basketball and football players. Previous research suggests that relative age is not a salient influence of the likelihood of reaching either the NBA or NFL [31, 32]. The fact that relative age did not influence career length in the current study provides further support that relative age is not relevant to the development of expertise in those sports (at least in North America).

Unlike the results for basketball and football, the significant relationship between relative age and career length in ice hockey supports the notion that relative age is an important constraint on the development of expertise in ice hockey. However, the results also reinforce the emerging evidence that the influence of relative age on development is complex. While relatively older players appear to experience advantages as youth, it appears that relatively younger athletes experience some advantages long term: Results from Table 1 suggest that relatively younger ice hockey players play approximately 100 more games than relatively older players (i.e. Q4 vs. Q1). This amounts to a little over one full season’s worth of games in NHL. Our results are therefore consistent with previous findings that relatively younger athletes may develop into more successful athletes [27, 30].

The reason that relatively younger players have longer careers is not known. It is possible that injuries influence an athlete’s career length. Baker et al. [40] hypothesized across sports that athletes who suffer from injuries drop out of professional sports early even though they have the potential to perform on a high level. As relatively younger individuals may be at a lower risk of injury as it was shown in Canadian youth ice hockey [9], they may have a longer career since they are at a lower risk of dropout due to injury. Earlier studies have shown relatively older players retire earlier from their sporting careers [14]. Collectively, these studies and the results from the current investigation strongly support that relative age influences athletes’ career length in North American professional hockey.

While our results mirror trends noted by others (e.g. [30]), we see some important distinctions emerging from the current results. Our third hypothesis was that in sports with a RAE, relatively younger players would play more matches at the highest levels of achievement than relatively older players. Expanding the consideration of career length from ‘games played’ to include a wide range of scores using the ‘ranks’ (Fig. 2a–c) may be important. Because the sample sizes are larger for quartiles 1 and 2 than those for quartiles 3 and 4 (see Fig. 1), it is not surprising that the range of ranks is considerably larger for quartiles 1 and 2 (range Q1/Q2 = 1 to 243; range Q3/Q4 = 1 to 133). On the one hand, the mean number of games played is greater for quartiles 3 and 4. On the other hand, the extent to which the trend of longer careers for relatively younger athletes (more games played) is viable or a statistical artefact may be worth considering. Visual inspection of Fig. 2c (NHL) suggests there is little distinction between quartiles 1, 2, 3 and 4 in terms of games played for ranks 1–35 (approximately). From ranks 35 to 125 (approximately), however, it appears that quartiles 1 and 2 played more games than their quartile 3 and 4 counterparts. Is the higher average of games played (Table 1) for quartiles 3 and 4 the result of greater talent in these athletes or a longer right tail of players from quartiles 1 and 2 playing fewer games (<200)?

Like any research, the current study had some limitations. For example, while the sampling frame for the current study (drafted athletes from 1980 to 1989) ensures that the results were not influenced by still-active players, the current study did not account for athletes who entered the North American professional sports leagues through non-entry draft pathways. While non-drafted professional athletes are certainly a small subgroup, it may be interesting to include such athletes and their career statistics in future research. In addition, there are a number of factors that influence career length in some sports [40]. For example, in the NFL, career length appears to vary by playing position (with quarterbacks having the longest careers). Going forward, it may be necessary to explore interactions between relative age and playing position on career length. It may also be important for future research to consider the different high performance pathways within the sports and any potential influence that may have on relative age-related outcomes. For example, most professional football and basketball players go through the college sport system, whereas hockey players typically play in the Canadian amateur developmental system. Last, it is possible that the trends observed in the current study are not generalizable to more recent cohorts of athletes. Further research will be required to address these issues and assess their impact on career length.

## Conclusions

Going forward, it will be important to establish how constraints on sport expertise, like relative age, influence the development of expertise longitudinally. We have estimates of how relative age influences selection in youth stages of participation, elite amateur stages and the elite professional levels, but it is difficult to infer the cause of trends identified at elite professional levels without a better sense of relative age’s influence throughout the process of athlete development. A detailed understanding of how relative age affects athlete outcomes across the entire process of athlete development would be valuable for determining the short- and long-term implications of this systemic bias in sports.
